# *Amorphophallus konjac*: A Novel Alternative Flour on Gluten-Free Bread

**DOI:** 10.3390/foods10061206

**Published:** 2021-05-27

**Authors:** Fernanda Laignier, Rita de Cássia Coelho de Almeida Akutsu, Iriani Rodrigues Maldonade, Maria Teresa Bertoldo Pacheco, Vera Sônia Nunes Silva, Marcio Antônio Mendonça, Renata Puppin Zandonadi, António Raposo, Raquel Braz Assunção Botelho

**Affiliations:** 1 Department of Nutrition, College of Health Sciences, University of Brasília, Brasília 70910900, Brazil; felaignier@hotmail.com (F.L.); rita.akutsu@gmail.com (R.d.C.C.d.A.A.); renatapz@unb.br (R.P.Z.); 2 Brazilian Agricultural Research Corporation (EMBRAPA-Hortaliças), Brasília 70359970, Brazil; irianimaldonade@embrapa.br; 3 Institute of Food Technology, São Paulo 13070178, Brazil; mtb@ital.sp.gov.br (M.T.B.P.); vera.silva@ital.sp.gov.br (V.S.N.S.); 4 College of Agronomy and Veterinary Medicine, University of Brasília, Brasília 70910900, Brazil; marcioamen@gmail.com; 5 CBIOS (Research Center for Biosciences and Health Technologies), Universidade Lusófona de Humanidades e Tecnologias, Campo Grande 376, 1749-024 Lisboa, Portugal

**Keywords:** gluten-free, bread, *Amorphophallus konjac*, baking

## Abstract

The demand for gluten-free products is rising, but their production with similar quality as their gluten counterparts is challenging. This study aimed to develop gluten-free bread samples using different concentrations of *Amorphophallus konjac* flour (0%, 12.5%, 25%, 37.5%, and 50% of the total flour content) and to evaluate their nutritional and physicochemical properties. Proteins, lipids, carbohydrates, moisture, ash content, fibers, resistant starch, firmness, specific volume, and color were evaluated using official methods. Protein varied from 2.95% to 4.94%, the energy value from 347.93 to 133.55 kcal/100 g, dietary fiber from 8.19 to 17.90%, and resistant starch from 0.67% to 0.75% on wet basis. The addition of konjac flour positively influenced the specific volume. Higher concentrations of konjac flour in the formulations led to lower calories of the bread due to the significant addition of water to the dough. The bread samples with konjac showed high fiber content due to the composition of the flour. They had lower levels of carbohydrates, which can positively influence the glycemic index. Konjac flour provided dough mold, growth, and better texture for gluten-free bread. The best formulations were prepared in concentrations up to 37.5% konjac. The 50% konjac bread showed slightly reduced specific volume and pale color.

## 1. Introduction

Due to the growing trend in consuming gluten-free products, the food industry has sought to expand and diversify its production to meet this increasing demand [1]. The gluten-free diet (GFD) has become popular since it is the only treatment for those who suffer from gluten-related disorders (GRD) [2,3], and their relatives consume gluten-free products to support the treatment and avoid food cross-contamination. Moreover, some individuals without GRD have adhered to GFD, believing in GFD’s potential health benefits, despite the lack of scientific evidence on it [2,4,5,6,7]. Therefore, about 10% of the world population has adopted a GFD [8,9,10,11]. In this sense, the gluten-free food market is expected to grow between 2019 and 2025, from US$ 3.73 billion to US$ 6.43 billion worldwide [12].

Among the gluten-free foods that make up the market’s largest share are bakery products [12], bread being the most desired product by GRD people. However, producing gluten-free bread with similar characteristics to the traditional product becomes a technological challenge due to the absence of proteins forming the gluten network, with elasticity and extensibility [8,13,14]. The absence of gluten in bread formulations, for example, results in some qualitative problems, low volume, brittle texture, undesirable taste, and short shelf life [15]. 

Foste et al. [16] claim that gluten-free products are high in starch and low in some nutrients, and fiber and strategies are needed to balance them. Soluble fibers as beta-glucan, chitosan, psyllium, and glucomannan have been studied as potential application in the gluten-free bakery market with potentially positive effects on consumers’ health [17,18]. Glucomannan is extracted from the tuber *Amorphophallus* *konjac* (a perennial plant from the subtropical regions of South East Asia and Africa). It is used in Chinese medicine for detoxification, cancer suppression, stasis of blood, treatment of asthma, cough, hernia, breast pain, and hematological and skin disorders [19]. Due to its water absorption capacity and stability, it is considered a source of hydrocolloidal dietary fiber. It has been used as a supplement to treat and prevent excess weight and diabetes and dermatological conditions [20].

As a food additive, glucomannan was tested in bakery products, drinks, bread, and pasta [19,21], but the flour of *Amorphophallus konjac* was not broadly studied. The flour from *Amorphophallus konjac* is considered a functional ingredient [22] containing about 1.4–3.4% of proteins, 78–80% of fibers, 8% of starch, and 1.7–2.1% of ash content [23,24,25]. This flour shows important health benefits in reducing cholesterol and triglycerides, improving blood sugar levels and promoting intestinal activity and human immune function. These health benefits potentially contribute to GRD individuals’ health and can be considered a potential healthy ingredient in gluten-free products [22]. Until now, no study used konjac flour as a substitute for wheat flour but as a food additive to improve gluten-free bread characteristics. Nakamura et al. [26] used *Amorphophallus konjac* flour in concentrations of 0.25%, 0.50%, and 0.75% as a thickener in gluten-free bread, and Moore et al. [27] used konjac flour at 1.5% added to 0.9% of xanthan in bread production. 

Considering the potential application of *Amorphophallus konjac* flour and its potential benefits to individuals suffering from GRD, this study aimed to develop gluten-free bread with *Amorphophallus konjac* flour and to evaluate the nutritional and physicochemical properties of the formulations with different concentrations of the flour. 

## 2. Materials and Methods

This experimental study took place in the Dietetic Laboratory from the University of Brasilia (Brazil) for bread production. For chemical composition analysis, color, texture, and specific volume, research was conducted in the Food Analysis laboratory (UnB), EMBRAPA, and ITAL (Food Technology Institute). All samples were developed and analyzed in triplicate.

### 2.1. Bread Preparation 

*Amorphophallus konjac* flour was purchased from a Brazilian pharmaceutical company (SM pharmaceutical enterprises) in three different lots (lots: 18F13-B022-034221, 18F13-B022-034226, MW20171011-1750) imported from China in packages of 1000 g each. These three flours were mixed in the exact amounts to prepare the formulations. The used flours were composed of 70% glucomannan konjac, according to the labels. They were previously microbiologically tested (total count of bacteria, mold and yeasts, *Escherichia coli*, and *Salmonella*) and approved by the Brazilian sanitary legislation. 

The control gluten-free bread (GFB) was composed of potato starch (30%) and rice flour (70%) as flour basis, added sucrose (12 g/100 g of control flour basis); salt (3 g/100 g of control flour basis); water (34.5 g/100 g of control flour basis); soy oil (16.5 g/100 g of control flour basis); whole egg (29.5 g/100 g of control flour basis); and yeast (1.5 g/100 g of control flour basis). This formulation was adjusted from the bread formulation studied by Aguiar [28], and no additives were used. The same ingredients were used in the modified GFB samples with the addition of konjac flour (Table S1 in Supplementary File). For the other samples, *Amorphophallus konjac* flour was added in the proportion of 12.5%, 25%, 37.5%, and 50% of the flour quantity. According to the literature, konjac flour presents almost 80% of fibers and only 8% of starch, making it unfeasible to use it above the maximum percentage applied in this study. Preliminary tests were performed with percentages superior to 50%, and bread samples presented a strong odor and “taste of fish”. For the samples added of konjac flour, the amount of sucrose, salt, soy oil, and eggs was the same as the control sample (considering the weight of the control flour basis). The amount of water was adjusted due to konjac flour’s fiber content that can absorb 200 times their weight in water [29]. The amount of water was previously tested (every 5 mL until a moldable dough was achieved), and the final water amount was determined in each sample based on the dough’s characteristics. Therefore, in the konjac flour samples, we used water in a concentration of 131%, 228%, 297%, and 406%, respectively, based on the weight of the control flour basis. The ingredients besides the konjac flour were purchased from local stores in the Federal District, Brazil.

After weighing ingredients, the yeast was pre-activated in sucrose and warm water (38 °C) for 10 min. Separately, rice flour, potato starch, konjac flour, and salt were mixed. Eggs, water, and oil were added to the dry ingredients. Finally, activated fresh biological yeast was added, and the dough was homogenized. This dough was kneaded and rested for 50 min, and then, it went through a second kneading and modeling (40 g spheres). We used a preheated gas oven Brastemp^®^-Brazil, at 180 °C to bake all the samples. On the day of the analysis, samples were prepared and baked. 

The cooking factor for each sample of bread was determined using the formula proposed by Araujo et al. [30].

(1) $$\mathrm{Cooking}\;\mathrm{Factor}\;=\frac{\mathrm{Baked}\;\mathrm{bread}\;\left(g\right)}{\mathrm{Bread}\;\mathrm{dough}}$$

Moreover, we calculated the weight loss percentage [31] after baking using the formula:

(2) $$\mathrm{Weight}\;\mathrm{loss}\;\mathrm{after}\;\mathrm{baking}\;=\frac{\mathrm{Dough}\;\left(g\right)\times 100}{\mathrm{Baked}\;\mathrm{bread}\;\left(g\right)-100}$$

### 2.2. Chemical Characterization

The Adolfo Lutz Institute’s analytical standards [32] were used to determine the moisture by the loss of water by drying, direct drying in the oven at 105 °C, followed by weighing the dry sample until constant weight. The determination of crude protein was performed using the official Kjeldahl method with adaptations. The samples went through the stages of digestion of organic matter, the distillation of nitrogen with the formation of ammonium hydroxide, and titrated directly with HCl 0.1 N. This resulted in a percentage of nitrogen calculated according to the volume spent on HCl, multiplied by the general factor of protein following the Association of Official Analytical Chemists (AOAC 991.22) [33]. The Am 5-04 method was used to determine lipid content, using the XT15 extractor (Ankom, Macedon, NY, US) from Ankom Technology, carried out by extraction with petroleum ether, by dragging, under pressure [34]. The ash content of the dry samples was determined by the incineration residue obtained from heating in a muffle furnace at 600 °C using a heating ramp of 240 min, according to method 945.45 [33]. Total dietary fiber (TDF) was evaluated by the enzymatic-gravimetric method, which consists of gelatinization and partial hydrolysis of starch, followed by hydrolysis of part of proteins and residual starch. Its value is expressed after subtracting the analytical blank (AB) and the protein and mineral content determined in the residues [33]. The total carbohydrate content was determined by difference, subtracting from 100 the values found for moisture, protein content, lipids, ash, and total fibers, according to method 986.25 [33]. According to the AOAC 2002.02 method and the American Association of Cereal Chemists (AACC 32-40.01) method, the resistant starch content was determined using a commercially test kit (Megazyme International Ireland Ltd., Wicklow, Ireland).

### 2.3. Color Evaluation 

The evaluation of the color of the crust, crumb, and bottom of the bread samples was carried out in a spectrophotometer ColorQuestXE (HunterLab, Reston, VA, USA). We obtained from the Hunter system the values for the coordinates L* (measurable in terms of white to black intensity), a* (measurable in terms of intensity from red to green), and b* (measurable in terms of yellow and blue intensity). It was possible to obtain hue angle h* (Equation (3)), color saturation or chroma C* (Equation (4)), and color difference ΔE (Equation (5)) [35,36,37,38]. L_0_, a_0_ and b_0_ are the coordinates obtained for the control sample.

(3) h∗=arctang (b∗/a∗)

(4) $$C*=\sqrt{\left(a{*}^{2}+b^{*2}\right)}$$

(5) $$\Delta E=\sqrt{\left[{\left(L*-L{*}_{0}\right)}^{2}+{\left(a*-a_{0}\right)}^{2}+{\left(b*-b_{0}\right)}^{2}\right]}$$

### 2.4. Texture Analysis

The texture profile (TPA) analysis of the bread samples was performed using the method 74-09.01—*Measurement of Bread Firmness by Universal Testing Machine* [39]. The equipment used was the TA.XTplus connected to the Software Exponent (version 6.1.4., Stable Micro System, Surrey, UK) A 36 mm cylindrical probe (Stable Micro System, Surrey, UK) was used, test speed 1.7 mm/s; deformation level of 40%, trigger load 5 g. This probe is usually used for bread samples. Bread samples were evaluated after baking and standing two hours out of the oven for cooling. They were tested as baked in small spheres (balls) like brioche bread. Data are expressed as the force necessary to deform the product as the cylindrical probe enters in contact with the bread.

### 2.5. Specific Volume

Specific volume was measured by the rapeseed displacement method [40] through the ratio between volume (cm^3^) and mass (g) of each sample. 

### 2.6. Total Energy Value 

The total energy value was reached with the macronutrients from the proximate composition analysis and the Atwater factors multiplying fats by 9 kcal/g, proteins by 4 kcal/g, and carbohydrates by 4 kcal/g [41]. As mentioned in Section 2.2, carbohydrates were reached by subtracting from 100 the values found for moisture, protein content, lipids, ash, and total fibers.

### 2.7. Statistical Analysis

The results were subjected to one-way ANOVA followed by Tukey’s post hoc test, with the level of *p* < 0.05 considered significant. Statistical analyses were performed using software SPSS-IBM (24.0, IBM, Armonk, NY, USA) All analyses were conducted in triplicate.

## 3. Results

### 3.1. Preparation of Bread Samples and Cooking Quality

Table 1 presents some characteristics of the formulations in the cooking process. Cooking times and weight losses during baking were different depending on the moisture of the dough. Table S1 presents the different formulations.

### 3.2. Chemical Characterization

Table 2 presents the chemical composition of the different gluten-free bread formulations. All samples were baked simultaneously and taken to the food analysis laboratory after one hour to start all chemical analysis. Protein content was slightly reduced with konjac flour increase with the lowest and significant value for the 50% konjac flour bread (*p* = 0.008). Lipids reduced 26.1% when comparing the control bread and the 25% konjac, and 51.3% compared to the 50% konjac. There was a reduction of 38.1% between the control sample and the 50% konjac sample for carbohydrates. The 50% konjac bread presented 34.5 more times fiber than the control bread for dietary fibers. The total energy value (TEV) decreased by 61.6% between control and 50% konjac samples.

### 3.3. Specific Volume, Firmness and Color

The specific volume (SV) is a measure to verify the dough’s ability to expand and retain the gas during baking [42]. Table 3 presents the data of the SV and firmness of the control and konjac samples. A statistically significant difference (*p* < 0.05) was obtained for SV from the control and all konjac samples, with a higher volume with the increase of konjac. However, only 12.5% bread is different from other formulations, demonstrating that the increase in konjac above 25% did not affect the volume. Konjac flour provided more significant bread expansion, contributing to its texture. It was impossible to measure control bread’s firmness in the same conditions as the other bread samples. Control bread presented a very hard texture after baking since it was only prepared with potato starch and rice flour and no additives to improve texture. The cylindrical probe did not penetrate the sample because of its hardness; therefore, the equipment did not provide reading parameters.

The average values of Chroma-C*, hue angle-h*, and color difference for the different samples are in Table 4. The chroma is related to the color’s purity, and higher values indicate more intense colors [43].

Analyzing the results of color saturation for the bread crust, the control bread obtained the highest average value indicating a more intense color. The bread crumb from the control also obtained the highest average for color saturation, differing statistically (*p* < 0.05) from the others. It indicates that as the konjac flour increases, a change in the crumbs’ color also increased, decreasing the degree of saturation and, consequently, the loss in color purity. The highest color saturation value was found in the control bread’s crust, and the lowest in the breadcrumbs 37.5 and 50%.

## 4. Discussion

Bread is one of the most popular items in the customer’s purchase basket [44], reaching the worldwide average consumption of 18 kg/year per capita [45,46]. To provide similar products for GRD individuals who follow a GFD is until now a challenge, mainly in terms of physicochemical properties. Weight loss when baking is very pronounced in gluten-free bread due to the absence of gluten’s protein network. Cooking factor expresses the dough’s ability to retain the water added to it [42].

In our study, the weight loss varied between 17 (control bread) and 32.5% (37.5% and 50% konjac flour bread). Moore et al. [27] using konjac flour at 1.5% showed a weight cooking loss of 9.20%, lower than our results. Turkut et al. [47] obtained losses from 14.4 to 15.4%, and Zelada et al. [31] observed a loss ranging from 11.9 to 15.0%, both lower than our findings. Weight loss during cooking provides information mainly on the amount of evaporated water. However, it also represents the loss of organic material, such as fermented sugars released in the form of CO₂ [48]. The format in which the konjac bread was shaped is different from the bread samples in the mentioned studies. Konjac bread samples were molded into small spheres, and in other studies, they were shaped as loaves. According to Horstmann, Foschia, and Arendt [48], it is possible that bread samples with a larger surface area present high cooking loss. Our results point to higher losses as konjac flour is added to the formulation. However, for the doughs’ shape before baking, the amount of water added to the formulations was 980% higher than the control bread. The network formed in these doughs did not allow all the added water to be retained, even with the high fiber content. It was observed that bread samples with more added water were kept longer in the oven to present the crunchiest crust. Therefore, longer baking time led to higher water losses. 

The konjac bread samples had lower protein levels than the bread studied by other authors [28,49]. Wang et al. [50] stated that the incorporation of protein ingredients in gluten-free doughs could improve the sensory and nutritional quality of gluten-free bread, in addition to an increase in flavor. The addition of proteins helps in forming a network similar to gluten in wheat bread [13]. However, in this study, the only variation was the konjac flour and water content to evaluate the use of a product rich in fiber, forming a barrier to maintain volume and texture. 

The protein content found in 12.5% konjac bread (4.9%) was higher than the other konjac gluten-free bread samples (Table 2) and slightly higher than the average protein found in gluten-free bread evaluated by Cornicelli et al. (4.29%, wet basis) [51] and by Roman, Belorio and Gomez (3.91%, wet basis) [52]. The highest amount of protein in the 12.5% konjac bread could be explained by the lower amount of water necessary to achieve moldable dough than the other konjac GFB samples (Supplementary File—Table S1), as confirmed by the higher moisture content in this sample. Roman, Belorio, and Gomez [52] claim that 81% of commercial gluten-free bread in their study had added proteins and that even so, the protein content of these bread samples was lower than their gluten-free counterparts. 

Bread samples with 37.5% and 50% konjac had the lowest levels of lipids. When evaluating the lipid content on formulations based on rice flour, Saueressig, Kaminski, and Escobar [49] observed that the highest average was 3.80%, similar to bread with a higher concentration of konjac flour (3.59%) in this study. According to Brandão and Lucena [53], the fats added to the formulations improve the dough’s quality, increase its extensibility, and provide the softness of the crumb and a more pleasant flavor. Jamieson, Weir, and Gougeon [54] observed that industrialized gluten-free products had up to 1.3 times more fat than their gluten counterparts, significantly increasing the consumption of calories. The 12.5% and 25% konjac GFB samples presented lipid content of 8.13% and 5.59%, respectively, similar to the GFB produced by Jamieson, Weir, and Gougeon (6.8%) [54]. 

All konjac bread samples obtained lower average carbohydrates than studies of bread with and without gluten by Cornicelli et al. [51]; Jamieson, Weir, and Gougeon [54]; and Roman, Belorio, and Gomez [52]. According to Jamieson, Weir, and Gougeon [54], gluten-free products generally have higher sugar content than gluten bread. 

A significant difference (*p* < 0.05) was obtained for the moisture content comparing all samples. The highest content was for 50% konjac bread, making up for more than half of the baked bread’s weight, which justifies the lower energy value. Aguiar [28] produced gluten-free bread with sorghum flour, and the highest value for moisture was 53.24%. The moisture content of a product influences the choice of packaging, the form of storage, and its processing [32]. Parry [55] reports that the use of konjac flour in concentrations of 0.1% to 0.5% influenced the release of moisture in bread, sweets, and bakery products. Horstmann, Foschia, and Arendt [48] affirm that bread moisture reflects in crumb softness after baking. This moisture difference resulted in softer crumbs for the konjac bread samples, as presented by the texture profile data.

The higher the proportion of konjac flour, the lower the protein content, lipids, and carbohydrates. Conversely, the total dietary fiber increases, demonstrating that konjac flour provides 21.8 times more fiber than control bread. According to Parry [55], the fibers in konjac flour can reach 90% of its composition. This fiber (Glucomannan) has beneficial properties such as prebiotic action [25], reducing cholesterol, improving blood sugar levels, and promoting immune function that are essential health benefits to GRD individuals [22].

Regarding fiber, gluten-free bread with konjac obtained better results than those by Saueressig, Kaminski, and Escobar [49], with soluble (inulin) and insoluble (rice bran) fibers. In Saueressig, Kaminski, and Escobar study [49], the formulation that contained the highest percentage of fibers showed an average of 4.88%, lower than our study bread samples with konjac in which dietary fibers ranged from 8.19% to 17.9%. Thus, according to Brazilian legislation, bread with konjac can be classified as food with high fiber content (>6 g/100 g) [56].

The recommendation for daily fiber intake is from 30 to 38 g for men and 21 and 25 g for women [25], a challenging amount to achieve in a gluten-free diet that features low-fiber food. The amount of fiber in labels of gluten-free bread by Nascimento et al. [14] and Lerma et al. [57] was less than the average for the same products with gluten. The average values in gluten-free bread found by these authors were 0.7% and 3.61%, respectively, with low values considering the recommendation.

In the preparation of gluten-free bread, corn, rice, and potato starches are often used to replace wheat flour. However, these products are low in fiber, micronutrients, proteins, and generally have a higher glycemic index [58]. The glycemic response of carbohydrates may increase in gluten-free foods because the gluten protein network surrounds the starch granule, being difficult for amylase action, thus inhibiting its hydrolysis in the lumen of the small gut [2]. Pellegrini and Agostoni [2] and Foste et al. [16] suggest supplementing gluten-free bread with soluble fibers so that there is a reduction in the glycemic index in these bread samples.

Samples with 25%, 37.5%, and 50% konjac had higher resistance starch levels than control bread. Compared to the results obtained in the analysis of commercial gluten-free bread performed by Larretxi et al. [59], bread with konjac flour had a low content of resistant starch. Konjac bread 50% presented 0.70 g/100 g, less than the commercial bread with 3.6% [59]. Resistant starch has a physiological behavior similar to soluble fiber. Its positive effects range from the formation of short-chain fatty acids, due to the prebiotic effect, to the decrease in postprandial glycemia and insulinemia [25].

The energy value ranged between 347 kcal/100 g in control and 133.55 kcal/100 g in the 50% konjac. The average energy value of gluten-free bread by Cornicelli et al. [51] and Roman, Belorio, and Gomez [52] is superior to all bread prepared with konjac flour. A large amount of fiber can explain the low energy value of konjac bread. 

The higher was the proportion of konjac flour added to the formulations, the higher were the average moisture, total dietary fibers, and resistant starch. Inversely to this, smaller were the averages for proteins, carbohydrates, lipids, and energy value. These results show that the konjac flour influenced positively the formulations of bread, improving the amount of micronutrients and fibers. The humidity was higher among the konjac bread samples favoring the texture; however, the macronutrients’ averages were low due to the increase of fibers. 

The bread samples of Moore et al. [27], using 1.5% konjac flour registered 2.08 cm^3^/g of SV, similar to bread samples above 25% konjac flour in this study. Hager and Arendt [60] obtained values of 1.78 and 1.63 cm^3^/g in their rice and cornbread, respectively. Sandri et al. [61] obtained SV between 1.22 and 1.70 cm^3^/g in their bread based on rice flour. Djordjevic et al. (2019) obtained a variation between 1.52 to 3.97 cm^3^/g of bread prepared with corn flour with added fibers. Zelada et al. [31] obtained results from 2.41 to 2.92 cm^3^/g in bread samples of corn and rice flour. However, the bread samples with higher SV were not precisely those that presented lower firmness values, as stated by Moore et al. [62] and Sandri et al. [61], in which there is a direct relationship between low specific volume and bread hardness. The values found for konjac bread samples are close to the average values presented in other studies.

Djordjevic et al. [63] report that dietary fibers can interfere with the quality of gluten-free bread by improving viscosity, texture, volume, sensory characteristics, and shelf life due to their water-binding ability, gel-forming ability, effects fat mimetics, textural, and thickeners.

In formulations of gluten-free bread prepared with rice flour, Nakamura et al. [26] used konjac flour in concentrations of 0.25%, 0.5%, and 0.75% as a thickener. The bread samples’ SV increased with increasing amounts of konjac from 0.25% to 0.50%, but it decreased by 0.75%. These authors also observed that konjac significantly reduced the bread’s hardness resulting in softer bread than those only with rice flour.

Texture can be defined as the mechanical, geometric, and surface attributes of a perceptible product using instruments and sensory means [64]. The taste of food is the most observed attribute for its acceptance. However, the texture is the main attribute considered to reject it. Gluten-free bread is characterized by a low volume, crumbly texture, and cracked crust [58], making them unattractive. Table 3 shows that 12.5% bread had the highest average of firmness. According to Giannou and Tzia [65], hardness is the maximum force necessary to compress food between teeth. Bread with higher konjac content is softer and probably easier to chew. Turkut et al. [47] observed that bread with 25% quinoa flour obtained the lowest average instrumental hardness. The results of the instrumental hardness analysis carried out by Arcanjo [66] on his gluten-free rice bread ranged from 1830.28 g to 4587.56 g. Zelada et al. [31], for their gluten-free bread prepared with different hydrocolloids, presented values from 1717 g to 3868 g. Gluten-free bread well evaluated made with fibers from the coffee husk [67] obtained average hardness varying between 1560.75 g and 5585 g. 

According to Foste et al. [16], the structure of the gluten-free dough requires higher amounts of water that resembles cake dough. Due to the amount of konjac flour, the dough became consistent, and it was possible to mold it into spheres without difficulty. The bread samples added konjac flour obtained a more significant water addition in their formulations and obtained the greatest losses during baking. The losses did not compromise the bread samples’ specific volume, as an increase of volume was observed as the proportion of flour increased.

According to Turkut et al. [47], bread color is the result of chemical reactions between proteins and carbohydrates during the baking process. The Maillard reaction is a way of darkening food that occurs with these two components, high temperatures and under ideal pH conditions [68]. During the heating of foods, reducing amino acids and sugars trigger a complex cascade of reactions that results in the formation of brown substances called melanoidins that provide a more attractive color to these foods [55]. An insufficient amount of reducing sugars and low protein content collaborates for pale color that often occurs in gluten-free bread [69].

When analyzing the results of color saturation for the bread crust, the control bread obtained the highest average value, indicating a more intense color. The control bread crumb also obtained the highest average color saturation. This indicates that, as konjac flour was added to the formulations, the change in the crumb color also increased, decreasing the degree of saturation and, consequently, the loss in color purity. The highest color saturation value was found in the crust of the control bread, and the lowest color saturation in the crumb of the bread 37.5 and 50%.

The results for bread crust tonality in this study corroborate the results found in the bread studied by Messa et al. [1], which also showed a tendency towards yellow. At the bottom of the bread, there was no statistical difference for color shade. However, the bottoms from 25%, 37.5%, and 50% konjac had lower averages indicating a color trend towards red. When analyzing the color difference variable in the bread crust, it was observed that there was no statistical difference between the samples of konjac bread.

## 5. Conclusions

The weight loss during the baking of the different formulations was lower in the control bread. The moisture content varied between 23.9% and 51.54%. The ash content on konjac bread samples is similar to those found in gluten-free bread from other studies. However, the ash content has increased according to konjac flour addition. It shows that the flour has contributed to the increase of minerals in the composition of the bread. The bread samples with konjac flour showed low caloric values and high fiber contents due to the konjac flour composition.

Additionally, they had lower carbohydrate levels, which can positively influence these samples’ glycemic index, but more studies are necessary to evaluate it. Considering the color analysis, the most intense color was obtained in the control bread. As the konjac flour was added to the formulations, the purity of the color was reduced. Konjac flour can be a promising alternative in preparing gluten-free bread because it provided dough mold, growth, and better texture when used in gluten-free bread. The best formulations were prepared in concentrations of up to 37.5% konjac. The 50% konjac bread showed low values for macronutrients, but it was observed that its specific volume was slightly reduced. A limitation of our study is the lack of sensory analysis of the developed bread samples, and further studies are necessary to evaluate their acceptability by consumers.

## Figures and Tables

**Table 1:** Cooking characteristics of different formulations of gluten-free bread samples.

	Percentage of Konjac Flour in Bread Formulations				
	0	12.5	25	37.5	50
% of cooking weigh loss	17	20	27.70	32.50	32.50
Cooking factor	0.83	0.80	0.72	0.68	0.68
Cooking time (Minutes)	24	24	30	30	33

**Table 2:** Chemical composition of different formulations of gluten-free bread with and without konjac flour addition on a wet basis.

	Konjac Flour Percentage in Bread Formulations				
	0	12.5	25	37.5	50
Protein (g/100 g)	5.9 ± 0.37 ^a^	4.94 ± 0.27 ^b^	4.0 ± 0.41 ^c^	3.88 ± 0.16 ^c^	2.95 ± 0.41 ^d^
Lipid (g/100 g)	10.84 ± 0.19 ^a^	8.13 ± 0.14 ^b^	5.59 ± 0.12 ^c^	3.89 ± 0.64 ^d^	3.59 ± 0.09 ^d^
Carbohydrates (g/100 g)	56.70 ± 0.54 ^a^	37.21 ± 0.69 ^b^	30.73 ± 0.27 ^c^	31.81 ± 0.59 ^c^	22.37 ± 0.27 ^d^
Moisture (g/100 g)	23.90 ± 0.28 ^e^	39.98 ± 0.56 ^d^	46.88 ± 0.48 ^b^	43.97 ± 0.42 ^c^	51.54 ± 0.86 ^a^
Ash content (g/100 g)	1.85 ± 0.03 ^a^	1.56 ± 0.01 ^bcd^	1.64 ± 0.04 ^bcd^	1.54 ± 0.08 ^cd^	1.66 ± 0.00 ^bc^
Dietary Fibers (g/100 g)	0.82 ± 0.02 ^e^	8.19 ± 0.01 ^d^	11.16 ± 0.09 ^c^	14.92 ± 0.06 ^b^	17.90 ± 0.32 ^a^
Resistant Starch (g/100 g)	0.64 ± 0.09 ^c^	0.67 ± 0.04 ^c^	0.70 ± 0.02 ^b^	0.75 ± 0.07 ^a^	0.70 ± 0.01 ^b^
Energy value (kcal/100 g)	347.93 ± 1.6 ^a^	241.73 ± 2.8 ^b^	189.19 ± 2.1 ^c^	177.76 ± 3.7 ^d^	133.55 ± 1.8 ^e^

Means followed by the same letter within lines do not differ statistically *p* > 0.05. All the analysis were performed in triplicate.

**Table 3:** Specific volume and texture of bread formulations prepared with different concentrations of konjac flour.

	Konjac Flour Percentage in Bread				
	0	12.5	25	37.5	50
Specific Volume	1.44 ± 0.06 ^e^	1.61 ± 0.06 ^d^	1.96 ± 0.06 ^b^	2.10 ± 0.12 ^a^	2.05 ± 0.10 ^ab^
Firmness (g)	*	4505.00 ± 343.97 ^a^	2508.31 ± 40.94 ^b^	1469.53 ± 39.91 ^c^	2334.90 ± 77.05 ^b^

Means followed by the same letter within lines do not differ statistically *p* > 0.05. * It was not possible to read the control bread due to its firmness.

**Table 4:** Mean values of chroma (C*), tone color (h*), and color difference (ΔE*) of bread formulations prepared with different concentrations of konjac flour.

Konjac Flour (%)	Crust Color		
	C*	h*	ΔE*
0	34.39 ± 2.37 ^a^	78.14 ± 2.25 ^a^	--- **
12.5	33.22 ± 0.29 ^a^	69.65 ± 5.75 ^d^	5.86 ± 3.14 ^a^
25	24.53 ± 0.58 ^b^	70.52 ± 2.65 ^c^	7.17 ± 1.49 ^a^
37.5	23.10 ± 0.70 ^b^	74.43 ± 2.73 ^b^	7.77 ± 0.95 ^a^
50	21.85 ± 0.45 ^b^	73.76 ± 2.78 ^c^	8.54 ± 0.46 ^a^
	Crumb color		
	C*	h*	ΔE*
0	26.55 ± 0.56 ^a^	85.83 ± 0.24 ^a^	--- **
12.5	22.37 ± 0.15 ^b^	85.06 ± 0.23 ^b^	5.95 ± 0.44 ^c^
25	16.75 ± 0.86 ^c^	86.03 ± 0.29 ^a^	10.23 ± 0.71 ^b^
37.5	14.12 ± 0.70 ^d^	86.26 ± 0.30 ^a^	13.24 ± 0.60 ^a^
50	14.61 ± 0.51 ^d^	84.76 ± 0.12 ^b^	14.14 ± 0.93 ^a^
	Bottom color		
	C*	h*	ΔE*
0	32.56 ± 4.10 ^a^	60.35 ± 6.11 ^a^	--- **
12.5	31.32 ± 2.75 ^a^	60.25 ± 2.95 ^a^	3.88 ± 0.80 ^b^
25	24.84 ± 2.97 ^ab^	56.93 ± 4.59 ^a^	8.86 ± 2.87 ^ab^
37.5	20.41 ± 1.77 ^b^	59.71 ± 1.43 ^a^	11.35 ± 1.92 ^a^
50	20.44 ± 1.16 ^b^	57.46 ± 0.81 ^a^	11.06 ± 1.64 ^a^

Means followed by the same letter within columns do not differ statistically, *p* > 0.05. All the analysis were performed in triplicate. ** There is not a ΔE* for the control bread sample.

## Data Availability

The study did not report any data.

## Supplementary Materials

The following are available online at https://www.mdpi.com/article/10.3390/foods10061206/s1, Table S1: Different formulations of gluten-free bread with and without the addition of konjac flour.

## References

[1] Messa S., Nabeshima E.H., Montenegro F.M., Cruz C.L.d.C.V. Estudo da obtenção de pães de forma sem glúten à base de derivados de mandioca. Proceedings of the 11th Congresso Interinstitucional de Iniciação Científica—CIIC 2017.

[2] Pellegrini N., Agostoni C. (2015). Nutritional aspects of gluten-free products. J. Sci. Food Agric..

[3] Menga V., Amato M., Phillips T.D., Angelino D., Morreale F., Fares C. (2017). Gluten-free pasta incorporating chia (*Salvia hispanica* L.) as thickening agent: An approach to naturally improve the nutritional profile and the in vitro carbohydrate digestibility. Food Chem..

[4] Missbach B., Schwingshackl L., Billmann A., Mystek A., Hickelsberger M., Bauer G., König J. (2015). Gluten-free food database: The nutritional quality and cost of packaged gluten-free foods. PeerJ.

[5] Falcomer A.L., Santos Araújo L., Farage P., Santos Monteiro J., Yoshio Nakano E., Puppin Zandonadi R. (2018). Gluten contamination in food services and industry: A systematic review. Crit. Rev. Food Sci. Nutr..

[6] Machado J., Gandolfi L., Coutinho De Almeida F., Malta Almeida L., Puppin Zandonadi R., Pratesi R. (2013). Gluten-free dietary compliance in Brazilian celiac patients: Questionnaire versus serological test. Nutr. Clin. Diet. Hosp..

[7] Farage P., Zandonadi R.P., Ginani V.C., Gandolfi L., Nakano E.Y., Pratesi R. (2018). Gluten-free diet: From development to assessment of a check-list designed for the prevention of gluten cross-contamination in food services. Nutrients.

[8] Cross C. (2013). Gluten-free industry is healthy, but is the food?. CMAJ.

[9] Melini V., Melini F. (2019). Gluten-free diet: Gaps and needs for a healthier diet. Nutrients.

[10] Sapone A., Bai J.C., Ciacci C., Dolinsek J., Green P.H.R., Hadjivassiliou M., Kaukinen K., Rostami K., Sanders D.S., Schumann M. (2012). Spectrum of gluten-related disorders: Consensus on new nomenclature and classification. BMC Med..

[11] Gadelha de Mattos Y., Puppin Zandonadi R., Gandolfi L., Pratesi R., Yoshio Nakano E., Pratesi C. (2019). Self-Reported Non-Celiac Gluten Sensitivity in Brazil: Translation, Cultural Adaptation, and Validation of Italian Questionnaire. Nutrients.

[12] STATISTA (2018). Global Gluten-Free Food Market Value from 2019 to 2025 (in Billion U.S. Dollars). https://www.statista.com/statistics/248467/global-gluten-free-food-market-size/#:~:text=The%20global%20market%20for%20gluten,to%208.3%20billion%20U.S.%20dollars.

[13] Houben A., Höchstötter A., Becker T. (2012). Possibilities to increase the quality in gluten-free bread production: An overview. Eur. Food Res. Technol..

[14] Bagolin do Nascimento A., Fiates Rataichesck Medeiros G., Teixeira E. (2013). Availbility, cost and nutitional composition of gluten-free products. Br. Food J..

[15] Pszczola D.E. (2012). The rise of gluten-free. Food Technol..

[16] Foste M., Verheyen C., Jekle M., Becker T. (2019). Fibres of milling and fruit processing by-products in gluten-free bread making: A review of hydration properties, dough formation and quality-improving strategies. Food Chem..

[17] Queiroz M.B., Nabeshima E.H. (2020). Naturalidade e Autenticidade. Brasil Bakery & Confectionery Trends.

[18] Zandonadi R.P., Botelho R.B.A., Araújo W.M.C. (2009). Psyllium as a Substitute for Gluten in Bread. J. Am. Diet. Assoc..

[19] Chua M., Baldwin T.C., Hocking T.J., Chan K. (2010). Traditional uses and potential health benefits of *Amorphophallus konjac* K. Koch ex N.E.Br. J. Ethnopharmacol..

[20] Choi K.H., Kim S.T., Bin B.H., Park P.J. (2020). Effect of *konjac glucomannan* (Kgm) on the reconstitution of the dermal environment against uvb-induced condition. Nutrients.

[21] Impaprasert R., Piyarat S., Sophontanakij N., Sakulnate N., Paengkanya S., Borompichaichartkul C., Srzednicki G. (2017). Rehydration and textural properties of dried konjac noodles: Effect of alkaline and some gelling agents. Horticulturae.

[22] Zannini E., Jones J.M., Renzetti S., Arendt E.K. (2012). Functional Replacements for Gluten. Annu. Rev. Food Sci. Technol..

[23] Li B., Xia J., Wang Y., Xie B. (2005). Grain-Size Effect on the Structure and Antiobesity Activity of Konjac Flour. J. Agric. Food Chem..

[24] Chen H.-L., Cheng H.-C., Liu Y.-J., Liu S.-Y., Wu W.-T. (2006). Konjac acts as a natural laxative by increasing stool bulk and improving colonic ecology in healthy adults. Nutrition.

[25] Li B., Shah B.R., Wang L., Liu S., Li Y., Wei X., Jin W., Li Z. (2015). Health benefits of konjac glucomannan with special focus on diabetes. Bioact. Carbohydr. Diet. Fibre.

[26] Nakamura R., Teshima Y., Miura M., Konishi F. (2016). Effect of Adding Glucomannan on the Rheological Properties, Sensory Characteristics and Staling of Gluten-free Rice Bread. J. Home Econ. Jpn..

[27] Moore M.M., Schober T.J., Dockery P., Arendt E.K. (2004). Textural comparison of gluten-free and wheat-based doughs, batters and breads. Cereal Chem..

[28] Andrade de Aguiar L., Bobrowski Rodrigues D., Vieira Queiroz V.A., Melo L., de Lacerde de Oliviera Pineli L. (2020). Comparison of two rapid descriptive sensory techniques for profiling and screening of drivers of liking of sorghum breads. Food Res. Int..

[29] Canga A.G., Martínez N.F., Sahagún A.M., Vieitez J.J.G., Liébana M.J.D., Pardo Á.P.C., Robles L.J.C., Sierra M. (2004). Glucomanano: Propiedades y aplicaciones terapéuticas. Nutr. Hosp..

[30] Araújo W.M.C., Botelho R.B.A., Pilla N.M., Borgo L.A. (2014). Alquimia dos Alimentos.

[31] Encina-Zelada C.R., Cadavez V., Teixeira J.A. (2019). Bread by a Mixture Design of Xanthan, Guar, and Hydroxypropyl Methyl Cellulose Gums. Foods.

[32] Izidoro D.R., Scheer A.d.P., Negre M.F.d.O., Haminiuk C.W.I., Sierakowski M.-R. (2008). Avaliação físico-química, colorimétrica e aceitação sensorial de emulsão estabilizada com polpa de banana verde. Rev. Inst. Adolfo Lutz.

[33] AOAC (2012). Official Methods of Analysis of the Association of Official Analytical Chemists.

[34] AOCS (2004). Approved Procedure Am 5-04 Rapid Determination of Oil/Fat Utilizing High Temperature Solvent Extraction.

[35] Little A. (1975). A research note off on a tangent. J. Food Sci..

[36] Mclellan M.R., Lind L.R., Kime R.W. (1994). Hue Angle Determinations and Statistical. J. Food Qual..

[37] Maskan M. (2001). Drying, shrinkage and rehydration characteristics of kiwifruits during hot air and microwave drying. J. Food Eng..

[38] Francis F.J. (1975). The origin of tan-1 a/b. J. Food Sci..

[39] American Association of Cereal Chemists (1999). AACC 74-09.01: Measurement of Bread Firmness by Universal Testing Machine.

[40] AACC (2000). Approved Methods of the American Association of Cereal Chemists.

[41] Ribeiro P., de Morais T.B., Colugnati F.A.B., Sigulem D.M. (2003). Tabelas de composição química de alimentos: Análise comparativa com resultados laboratoriais. Rev. Saude Publica.

[42] Evangelho J.A., Pinto V.Z., Zavareze R.E., Vanier L.N., Dias A.R.G., Barbosa L.M.P.B. (2012). Propriedades tecnológicas e nutricionais de pães preparados com diferentes proporções de farinha de arroz e farinha de arroz extrusada. Rev. Bras. Agrocienc..

[43] Jacomino A.P., Mendonça K., Kluge R.A. (2003). Armazenamento refrigerado de limões “Siciliano” tratados com etileno. Rev. Bras. Frutic..

[44] FMCG NEWS GLOBAL (2018). Global Bread and Bakery Consumption Continues to Experience Modest Growth. https://www.bizcommunity.com/Article/1/162/176273.html.

[45] Eglite A., Kunkulberga D. Foodbalt 2017 Bread Choice and Consumption Trends. Proceedings of the 11th Baltic Conference on Food Science and Technology “Food Science and Technology in a Changing World”.

[46] The World Bank Global consumption database: Bread. http://datatopics.worldbank.org/consumption/product/Bread.

[47] Turkut G.M., Cakmak H., Kumcuoglu S., Tavman S. (2016). Effect of quinoa flour on gluten-free bread batter rheology and bread quality. J. Cereal Sci..

[48] Horstmann S.W., Foschia M., Arendt E.K. (2017). Correlation analysis of protein quality characteristics with gluten-free bread properties. Food Funct..

[49] Saueressig A.L.C., Kaminski T.A., Escobar T.D. (2016). Inclusion of dietary fiber in gluten-free breads. Braz. J. Food Technol..

[50] Wang K., Lu F., Zhao L., Han C. (2017). Recent developments in gluten-free bread baking approaches: A review. Food Sci. Technol..

[51] Cornicelli M., Saba M., Machello N., Silano M., Neuhold S. (2018). Nutritional composition of gluten-free food versus regular food sold in the Italian market. Dig. Liver Dis..

[52] Roman L., Belorio M., Gomez M. (2019). Gluten-Free Breads: The Gap Between Research and Commercial Reality.

[53] Brandão S.S., Lira H.d.L. (2011). Tecnologia de Panificação e Confeitaria.

[54] Jamieson J.A., Weir M., Gougeon L. (2018). Canadian packaged gluten-free foods are less nutritious than their regular gluten-containing counterparts. PeerJ.

[55] Parry J.M. (2010). Konjac Glucomannan. Food Stabilizers, Thickeners and Gelling Agents.

[56] Ministério da Saúde—Agência Nacional de Vigilância Sanitária (2012). RDC No 54, de 12 de Novembro de 2012.

[57] Calvo-Lerma J., Crespo-Escobar P., Martínez-Barona S., Fornés-Ferrer V., Donat E., Ribes-Koninckx C. (2019). Differences in the macronutrient and dietary fibre profile of gluten-free products as compared to their gluten-containing counterparts. Eur. J. Clin. Nutr..

[58] Capriles V.D., Arêas J.A.G. (2014). Novel Approaches in Gluten-Free Breadmaking: Interface between Food Science, Nutrition, and Health. Compr. Rev. Food Sci. Food Saf..

[59] Larretxi I., Churruca I., Navarro V., Miranda J., Lasa A., Bustamante M.A., Simon E. (2019). Impact of analytically measured fiber and resistant starch from gluten free products on celiac peoples diet. J. Pre Proof.

[60] Hager A.S., Arendt E.K. (2013). Influence of hydroxypropylmethylcellulose (HPMC), xanthan gum and their combination on loaf specific volume, crumb hardness and crumb grain characteristics of gluten-free breads based on rice, maize, teff and buckwheat. Food Hydrocoll..

[61] Sandri L.T.B., Santos F.G., Fratelli C., Capriles V.D. (2017). Development of gluten-free bread formulations containing whole chia flour with acceptable sensory properties. Food Sci. Nutr..

[62] Moore M.M., Heinbockel M., Dockery P., Ulmer H.M., Arendt E.K. (2006). Network formation in gluten-free bread with application of transglutaminase. Cereal Chem..

[63] Djordjević M., Šoronja-Simović D., Nikolić I., Djordjević M., Šereš Z., Milašinović-Šeremešić M. (2019). Sugar beet and apple fibres coupled with hydroxypropylmethylcellulose as functional ingredients in gluten-free formulations: Rheological, technological and sensory aspects. Food Chem..

[64] Esteller M.S., Amaral R.L., Da Silva Lannes S.C. (2004). Effect of sugar and FAT replacers on the texture of baked goods. J. Texture Stud..

[65] Giannou V., Tzia C. (2007). Frozen dough bread: Quality and textural behavior during prolonged storage—Prediction of final product characteristics. J. Food Eng..

[66] Arcanjo F.M. (2017). Development and Characterization of Gluten-Free Bread Enriched with Maca (*Lepidium meyenii*), Canary Seed (*Phanaris canariensis*) and Niger (*Guizotia abyssinica*) flours. Master’s Thesis.

[67] Rios M.B., Iriondo-Dehond A., Iriondo-Dehond M., Herrera T., Dolores M. (2020). Effect of Coffee Cascara Dietary Fiber on the Physicochemical, Nutritional and Sensory Properties of a Gluten-Free Bread Formulation. Molecules.

[68] de Oliveira F.C., Coimbra J.S.d.R., de Oliveira E.B., Zuñiga A.D.G., Rojas E.E.G. (2016). Food Protein-polysaccharide Conjugates Obtained via the Maillard Reaction: A Review. Crit. Rev. Food Sci. Nutr..

[69] van Riemsdijk L.E., Van der Goot A.J., Hamer R.J., Boom R.M. (2011). Preparation of gluten-free bread using a meso-structured whey protein particle system. J. Cereal Sci..

