# Supplementary material for: Amorphophallus konjac: A Novel Alternative Flour on Gluten-Free Bread
Source: Foods. 2021 May 27;10(6):1206. doi: 10.3390/foods10061206 (PMC8229984; doi:10.3390/foods10061206)
Supplement: Supplementary file 1 [file foods-10-01206-s001.zip › foods-1232670-supplementary.pdf]

Table S1. Different formulations of gluten-free bread with and without the addition of konjac flour.

Ingredients		Control Bread	Bread 12.50%*	Bread 25%*	Bread 37.50%*	Bread 50%*
**Control flour basis**	Potato starch (g)	30	30	30	30	30
	Rice flour (g)	70	70	70	70	70
Konjac flour (g/100g of control flour basis)		0	14.3	33.3	60	100
Crystal sugar (g/100g of control flour basis)		12	12	12	12	12
Salt (g/100g of control flour basis)		3	3	3	3	3
Water (g/100g of control flour basis)		34.5	131	228	297	406
Soy oil (g/100g of control flour basis)		16.5	16.5	16.5	16.5	16.5
Whole Egg (g/100g of control flour basis)		29.5	29.5	29.5	29.5	29.5
Yeast (g/100g of control flour basis)		1.5	1.7	2	2.4	3

* The percentage value is related to the weight of the konjac flour in the total flour(potato starch + rice flour + konjac flour) amount.
